# Quantifying the Effect of Monitor Wear Time and Monitor Type on the Estimate of Sedentary Time in People with COPD: Systematic Review and Meta-Analysis

**DOI:** 10.3390/jcm11071980

**Published:** 2022-04-01

**Authors:** Fiona Coll, Vinicius Cavalheri, Daniel F. Gucciardi, Sheldon Wulff, Kylie Hill

**Affiliations:** 1 Curtin School of Allied Health, Faculty of Health Sciences, Curtin University, Perth, WA 6102, Australia; fiona.coll@postgrad.curtin.edu.au (F.C.); vinicius.cavalheri@curtin.edu.au (V.C.); d.gucciardi@curtin.edu.au (D.F.G.); 2 Physiotherapy Department, Royal Perth Hospital, Perth, WA 6000, Australia; sheldon.wulff@gmail.com; 3 Curtin enAble Institute, Faculty of Health Sciences, Curtin University, Perth, WA 6102, Australia; 4 Allied Health, South Metropolitan Health Service, Perth, WA 6150, Australia; 5 Exercise Medicine Research Institute, Edith Cowan University, Perth, WA 6027, Australia; 6 Institute for Respiratory Health, Sir Charles Gairdner Hospital, Nedlands, WA 6009, Australia

**Keywords:** COPD, inclinometer, meta-analysis, sedentary time, systematic review

## Abstract

In studies that have reported device-based measures of sedentary time (ST) in people with chronic obstructive pulmonary disease (COPD), we explored if the monitor type and monitor wear time moderated the estimate of this measure. Five electronic databases were searched in January 2021. Studies were included if >70% of participants had stable COPD, and measures of ST (min/day) were collected using wearable technology. Meta-regression was used to examine the influence of moderators on ST, monitor type, and wear time. The studies identified were a total of 1153, and 36 had usable data for meta-analyses. The overall pooled estimate of ST (mean [95% CI]) was 524 min/day [482 to 566] with moderate heterogeneity among effect sizes (I^2^ = 42%). Monitor wear time, as well as the interaction of monitor wear time and monitor type, were moderators of ST (*p* < 0.001). The largest difference (−318 min; 95% CI [−212 to −424]) was seen between studies where participants wore a device without a thigh inclinometer for 24 h (and removed sleep during analysis) (675 min, 95% CI [589 to 752]) and studies where participants wore a device with a thigh inclinometer for 12 h only (356 min; 95% CI [284 to 430]). In people with COPD, the monitor wear time and the interaction of the monitor wear time and the monitor type moderated the estimate of ST.

## 1. Introduction

Sedentary behaviour is defined as any behaviour undertaken during waking hours, in a seated or reclined posture, that requires low energy expenditure (i.e., <1.5 metabolic equivalents of tasks [METs] [1]. Common examples in older adults include television viewing, reading, completing crossword puzzles [1]. There is recognition that reducing sedentary time (ST) is an important lifestyle target for many clinical populations [2,3,4], including adults with chronic obstructive pulmonary disease (COPD) [5,6]. Specifically, in this population, increased ST has been linked with deleterious health outcomes such as a higher risk of cardiometabolic disease [7,8], and clinical trials are now reporting device-measured ST as an outcome of interest [9,10,11]. This review targets adults with chronic obstructive pulmonary disease (COPD), as this population is extremely sedentary [12] and is at greater risk of poor health outcomes due to pre-morbid health conditions [13] and engagement in prolonged, uninterrupted sitting [5,6]. 

The approach used to quantify ST in people with COPD differs considerably across studies [14]. That is, many studies have focused on measuring physical activity as the primary outcome and then have quantified daily inactivity (sitting, lying, and standing still) data as ST. This is achieved by downloading data collected with wearable devices and classifying the time during which movement was recorded as ‘physical activity’, and the time during which no movement was recorded as ‘ST’. That is, periods of physical inactivity are classified as ST [15]. This issue is overcome by using wearable devices that include an inclinometer on the thigh, which can separate inactivity into behaviour undertaken in seated or reclined posture (where the thigh is horizontal and should be classified as ST) from those undertaken when standing still (where the thigh is vertical and should be classified as light-intensity physical activity) [16].

Another factor that may have little influence on measures of physical activity but may produce large differences in the measure of ST is monitor wear time (e.g., 12 h vs. all waking hours). That is, in people with COPD, physical activity is most likely to occur during daylight hours, which will be captured over a 12 h sampling period (e.g., 07:00 to 19:00). In contrast, the time between sunset and going to bed for overnight sleep is very likely classified as ST (e.g., television viewing), and ceasing data collection in the early evening (e.g., 19:00) may mean ST will be grossly underestimated. 

To explore these issues and provide information on the precision of measures of ST, we undertook a systematic review and meta-analyses to address the following research question: In studies that have reported on device-measured ST in people with COPD, does monitor type (i.e., with or without a thigh inclinometer) or monitor wear time moderate the estimate of this measure?

## 2. Methods

### 2.1. Study Selection

This study has been reported in accordance with the Preferred Reporting of Items for Systematic Review and Meta-analysis (PRISMA) guidelines [17] (File S1). Studies were included if >70% of the participants had stable COPD, and measures of ST (min/day) were collected using wearable technology (e.g., accelerometers, inclinometers). Studies published only as conference abstracts or in a language other than English were excluded. 

### 2.2. Search Strategy

Studies were identified by searching five electronic databases: CINAHL, the Cochrane Library, EMBASE, (via OVID), PEDro (Physiotherapy Evidence Database), and PubMed from their inception to 7 January 2021. Reference lists (hand searches) from relevant papers were also screened. The search strategy used for PubMed was adapted for use in other databases (Figure S1).

The research question addressed in this systematic review was not included in the prospective registration of PROSPERO (CRD42019138106). The analyses presented in this paper constitute an additional analysis relating to the broad theme of sedentary behaviour and people with COPD.

Using Covidence software, [18] two review authors (FC and SW) independently screened titles, abstracts, and full papers as required to identify eligible studies. Disagreement was resolved by discussion or when needed by a third review author (KH). 

### 2.3. Data Extraction

Data were extracted into Microsoft Excel database by one author (FC) and checked by another author (KH). Data were extracted on sample size, monitor type, monitor wear time and, where appropriate, the method to omit sleep time from analysis. That is, it was noted whether the study asked participants to: (i) wear the monitor for a standard hours period (12 h protocol), (ii) remove the monitor overnight so that sleep was not included in the estimate of ST (waking hours protocol) or, (iii) wear the monitor continuously and during analysis, data that appeared to be overnight sleep were omitted (24 h sleep removed protocol). Where studies had collected measures of ST before and after an intervention, only data collected prior to the intervention were included in our analysis. The mean and standard deviation (SD) of the estimate of ST was expressed in natural units (i.e., min/day). If the estimates were reported using other measures of central tendency and dispersion (e.g., median and interquartile range), online software was used to derive estimates of mean and SD [19]. In the case of missing data, study authors were contacted via email on a maximum of three occasions.

### 2.4. Statistical Analysis

Included studies were coded and grouped accordingly to the moderator variables (i.e., monitor type and monitor wear time) into six groups: (i) *monitor type*: had a thigh inclinometer; *wear time*: 12 h; (ii) *monitor type*: had a thigh inclinometer; *wear time*: waking hours; (iii) *monitor type*: had a thigh inclinometer; *wear time*: 24 h with sleep removed; (iv) *monitor type:* no thigh inclinometer; wear time: 12 h; (v) *monitor type:* no thigh inclinometer; *wear time*: waking hours; (vi) *monitor type:* no thigh inclinometer; *wear time:* 24 h sleep removed. We analysed the data using random-effects, 3-level meta-analytic model via the package *metafor* [20] in the R statistical platform was used to account for dependencies of effects, namely, sampling variance of individual effects (level 1), as well as variance of effects within (level 2) and between (level 3) studies. The main and interaction effects of wear time and monitor type were estimated within random effects, meta-regression framework in which the null hypothesis is that the overall pooled effect is the same for all levels of the covariate [21]. 

## 3. Results

### 3.1. Study Selection and Grouping

**Figure 1:**
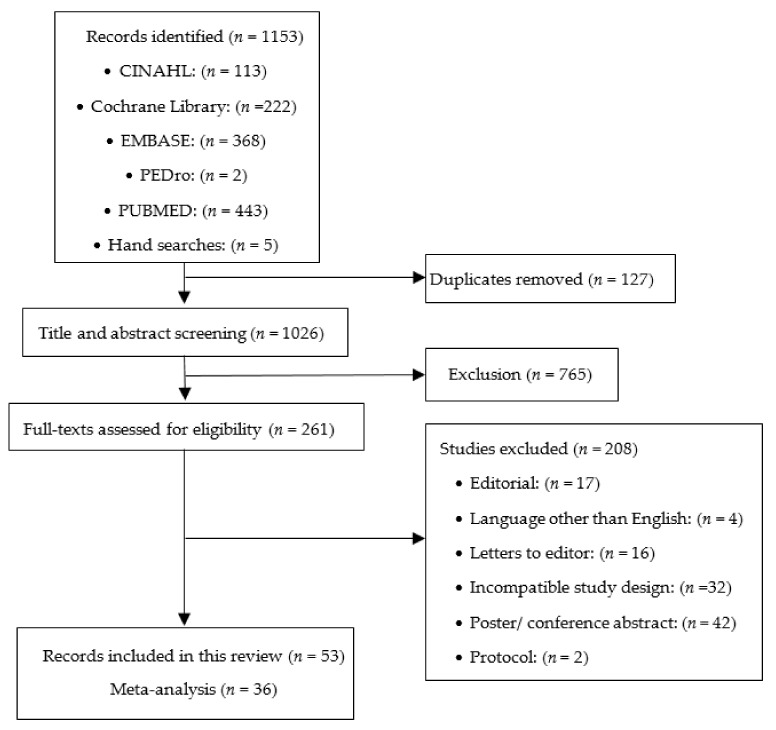
Flow of studies through the review.

The search yielded a total of 1153 records, of which 127 (11%) were duplicates. Of the remaining 1026 records, 765 (75%) were excluded during the title and abstract screening, and 208 (20%) were excluded following a full-text review (Figure 1). The Cohen’s Kappa for agreement regarding the inclusion of the studies between the two review authors was 0.98.

Of the remaining 53 studies, 36 reported data in a way that could be used in the meta-analyses. Of these, 25 (69%) were observational, and 11 (31%) were interventional studies. Regarding monitor type, 33% (*n* = 12) of the studies used a monitor that included a thigh inclinometer and was attached to the hip, upper arm, lumbar spine, or ankle. Regarding monitor wear time, 44% of studies (*n* = 16) used a 12 h protocol, 25% (*n* = 9) using a waking hours protocol, and 31% (*n* = 11) used a 24 h protocol.

All studies in this review measured physical activity as a primary outcome. Thirteen (36%) studies also described ST as a primary outcome, and the others reported ST as a secondary outcome.

### 3.2. Characteristics of Participants

The characteristics of 36 studies are presented in Table 1. Studies were conducted in Australia [6,9,10,11,22,23], Austria [24], Brazil [5,12,25,26,27,28,29,30,31,32,33,34,35,36,37], Canada [38], Germany [39]. Greece [40], Japan [41], Korea [42], the Netherlands [43,44], Portugal [45,46], Saudi Arabia [47], Sweden [48], United Kingdom [49], and the United States of America [50,51]. The total number of participants across 36 studies was 3914 (56% males), and the mean ± SD for age was 67 ± 8 years. The (mean ± SD) forced expiratory volume in one second (FEV_1_) ranged between 24 ± 9% and 85 ± 28% predicted, with the sample size of the included studies ranging between 10 and 941 participants.

### 3.3. Meta-Analysis

**Figure 2:**
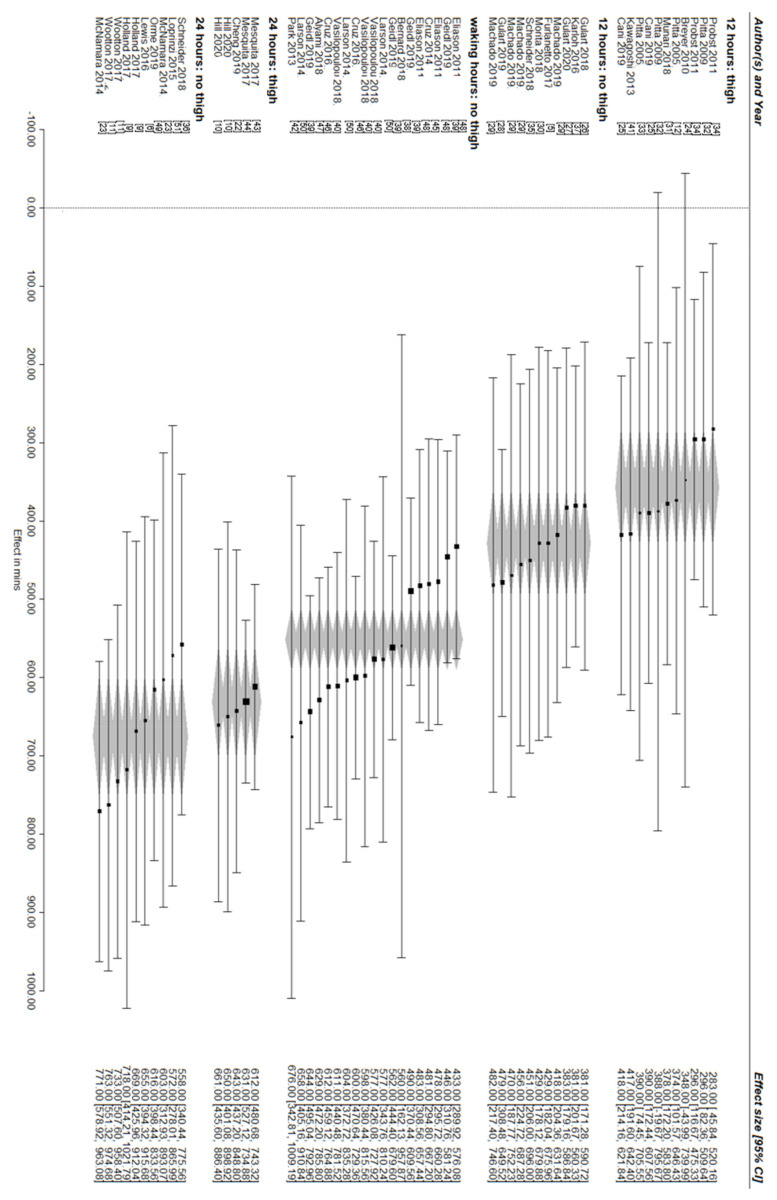
Forest plot of estimates of sedentary time with studies grouped according to wear time and monitor type. Data are represented as mean [95% confidence interval]. The grey diamonds represent the pooled 95% confidence interval for that category.

The overall pooled estimate of ST was 524 min/day (95% CI [482 to 566]), with a moderate amount of heterogeneity among effect sizes (I^2^ = 42%). Established guidelines for the interpretation of the proportion of total variance in effect estimates that is due to heterogeneity rather than sampling error are as follows: 0–40% = might not be important; 30–60% = may represent moderate heterogeneity; 50–90% = may represent substantial heterogeneity; and 75–100% = considerable heterogeneity [52]. Wear time (F (2,53)) = 26.23, *p* < 0.001 but not monitor type alone, (F (1,55)) = 2.21, *p* = 0.14) were meaningful moderators of the overall pooled estimate of ST. In terms of wear time, there was a stepwise reduction in ST between studies that used 24 h sleep removed protocol (651 min, 95% CI [599 to 703]), a waking hours protocol (551 min, 95% CI [512 to 590]) and a 12 h protocol (396 min, 95% CI [346 to 445]). For monitor type, the difference between no thigh inclinometer (545 min, 95% CI [494 to 596]) and thigh inclinometer (478 min, 95% CI [404 to 552]) was statistically inconsequential. When considering the interaction between wear time and monitor type, the highest ST was recorded by those studies that used ST in a 24 h sleep removed protocol and a device without a thigh inclinometer (675 min, 95% CI [598 to 752]), and the smallest ST was recorded by those studies which used a 12 h protocol and a device with thigh inclinometer (357 min 95% CI [284 to 430]) and the remaining estimates are between these quantities and are as follows: 12 h protocol and a device without a thigh inclinometer (429 min 95% CI [361 to 496]); waking hours protocol with a device without a thigh inclinometer (551 min 95% CI [512 to 590]); 24 h protocol with a device with a thigh inclinometer (631 min 95% CI [561 to 702]) (Figure 2) (Supplementary Material File S2: meta-analysis output file; Table S1 Data final).

## 4. Discussion

This systematic review with meta-analyses is the first to report the range of methodologies used by studies that collected device-based measures of ST in people with COPD. The main findings were that studies in this area used highly disparate approaches to measure ST and that monitor wear time and the interaction of monitor wear time and monitor type influenced the estimate of ST. 

Although understanding how people with COPD spend their waking hours has been an area of interest for more than a decade, an in-depth exploration of the methodological considerations regarding the measurement of ST in this population is lacking. This is because the focus of studies that have explored the use of waking hours in COPD has almost exclusively been on participation in physical activity. Specifically, in 2021 a task force comprised of researchers and key industry partners reviewed the data from the US-based COPDGene study [53] and the EU-based IMI-JU PROactive [54], as well as studies from individual consortium members that had collected measures of physical activity in COPD [55]. Based on their review of these data, a standardised methodology was proposed to guide the collection of device-measured physical activity in future research [55]. Recommendations were made regarding a minimum acceptable daily wear time of >8 h. Notwithstanding the well-established health benefits of regular participation in physical activity during daily life [56,57], epidemiological data collected in the general adult population have demonstrated that total ST in prolonged uninterrupted bouts increased the risk of cardiometabolic disease [2,5,58]. Of note, in people with COPD, similar associations have reported that reducing ST is increasingly considered an outcome for intervention-based studies [23,43]. Of note, although all studies included in the current review reported physical activity as a primary outcome, ST was also described as a primary outcome in 13 (36%) of studies. This highlights the need to understand the methodological considerations associated with reporting this outcome.

Our study is the first to demonstrate that monitor wear time is a moderator of the estimate of ST. That is, the longer the wear time protocol, the greater was the estimation of ST. For example, the mean difference in ST between studies that asked participants to wear devices for 24 h (and removed sleep during analysis) and those that asked participants to wear devices for 12 h was −318 min; (95% CI [−370 to −266]). Constraining monitor wear time to 12 h, which was the methodology adopted by 44% of the included studies, markedly reduced the potential for the true representation of ST. Conversely, the monitor type was not a moderator of ST. This is likely to reflect the discrepancy across the different subgroups. That is, studies that used a monitor that included a thigh inclinometer represent 6% of the combined sample size, whereas those studies which used a monitor that did not include a thigh inclinometer have 94% of the combined sample size.

Our study demonstrated that the interaction between monitor type and monitor wear time moderated ST. The smallest estimate of ST (356 min/day) was derived from studies that used a 12 h wear time protocol and a device that included a thigh inclinometer. This is because these studies underestimated ST with their short wear time protocol of 12 h. By using these data from a thigh inclinometer, we were also able to correctly classify standing still as light intensity physical activity rather ST; therefore, the estimate was smaller. The largest estimate of ST (675 min/day) was derived from studies that captured all waking hours using a 24 h wear time protocol (and removed sleep during analysis) and used a monitor without a thigh inclinometer, which would have misclassified standing still (inactivity) as ST, so, therefore, it was a larger estimate.

This review found that some of the studies used different anatomical locations for inclinometers. Although 25 percent of the studies included in this review used a monitor that incorporated an inclinometer attached to the upper arm, in this location, the inclinometer can only separate lying down (where the arm is horizontal) from sitting (the arm is vertical). Studies in other clinical populations [59,60,61,62] and that had no-clinical populations [16,63] have found similar results indicating the monitors with thigh inclinometers are less likely to misclassify standing as ST.

### Strengths and Limitations

The strengths of this systematic review include: the use of two independent assessors to determine study inclusion, multiple attempts to contact authors of studies to clarify their suitability for inclusion, accounting for dependency among effects in our statistical model (i.e., multiple effects from the same sample) and missing or unpublished outcome data. Nevertheless, we were unable to include data from all studies. We accept that the influence of monitor wear time may be less when ST is expressed as a percentage of total wear time. However, we were unable to perform a meta-regression on ST expressed as a percentage of total wear time as these measures were not consistently reported with dispersion measures. We also note that the Dynaport Activity Monitor (DAM) (McRoberts BV, The Hague, Netherlands) and the SenseWear Armband (Bodymedia Inc, SenseWear Professional, Pittsburgh, (USA) that were used in 15 (42%) studies included in the review are no longer commercially available. Finally, although the topic of this systematic review broadly falls within the scope of a larger program of research that was prospectively registered (PROSPERO; CRD42019138106 and Open Science Framework; httpps://bit.ly/3j8Dt3n; accessed on 27 August 2021).), the specific research questions addressed in this study review were not specifically stated in these documents [64].

## 5. Conclusions

This systematic review and meta-analysis demonstrated that when collecting measures of ST in people with COPD, monitor wear time and the interaction of the monitor wear time and monitor type influence the estimate. These data suggest a meaningful comparison of the estimate of ST between studies or between time-points within the study is only possible when both monitor type and monitor wear time have been standardised. These considerations may not be important when quantifying physical activity but highlight the unique nuances in measuring ST. Specifically, we recommend that ST is measured using monitors that use an inclinometer located on the thigh to allow standing (which is LIPA) and sitting (which is ST) to be **separated and properly** classified. Further, it seems most appropriate to measure ST over 24 h but ensure that sleep is removed during the analysis by asking participants to diarise sleep and/or the application of processing algorithms.

## Figures and Tables

**Table 1:** Characteristics of included studies.

Study (Publication Year)	Total Sample Size	Age, Year	Males, N (Y%)	FEV_1_, % Predicted	Device	Time over which Participants were Instructed to Wear the Device	Minimum Daily wear Time to be Included in Analyses	Minimum Number of Days Data Needed to be Available to be Included in Analyses	Days Used in Data Analyses
Studies which used a monitor with a thigh inclinometer and a 12 h protocol									
Breyer (2010) [24]	60	60 ± 9	27 (45)	46 ± 18	DAM	3 days	12 h	3 days	Weekend days were excluded
Cani (2019) [25]	59	O_2_: 68 ± 8 C: 67 ± 8	O_2_: 21 (79) C: Not recorded	O_2_: 25 ± 7 C: 24 ± 9	DAM	2 days	12 h	2 days	All days included
Kawagoshi (2013) [41]	26	77 ± 6	26 (100)	53 ± 26	A-MES	7 days	12 h	2 days	All days included
Munari (2018) [31]	115	66 ± 8	75(68)	35 ± 16	DAM	Not reported	12 h	2 days	All days included
Pitta (2005) [12]	50	64 ±7	36 (72)	43 ± 18	DAM	2 days	12 h	2 days	All days included
Pitta (2005) [33]	13	61 ±8	10 (77)	33 ± 10	DAM	1 day	12 h	1 day	All days included
Pitta (2009) [32]	80	A: 63 ± 7 B: 66 ± 8	A: 21 (53) B: 18 (45)	A: 48 ± 17 B: 46 ± 17	DAM	2 days	12 h	2 days	All days included
Probst (2011) [34]	40	Ix: 65 ± 10 C: 67 ± 7	21 (52)	Ix: 39 ± 14 C: 40 ± 13	DAM + SWA	2 days	12 h	2 days	Weekend days were excluded
**Studies that used a monitor without a thigh inclinometer and a 12 h protocol**									
Furlanetto (2017) [6]	101	66 (62–72) median (IQR)	58 (57)	41(30–50)	SWA + Dynaport	2 days	12 h	2 days	Weekend days were excluded
Gulart (2018) [26]	59	65 ± 9	45 (76)	35 ± 13	Dynaport minimod	2 days	12 h	2 days	All days included
Gulart (2020) [27]	53	64 ± 9	37 (70)	38 ± 14	Dynaport minimod	2 days	12 h	2 days	All days included
Gulart (2020) [28]	61	65 ± 9	47 (77)	35 ± 13	Dynaport minimod	2 days	12 h	2 days	All days included
Karloh (2016) [37]	38	65 ± 7	22 (58)	41 ± 15	Dynaport minimod	2 days	12 h	2 days	All days included
Machado (2019) [29]	270	G1: 67 ± 8 G2: 67 ± 8 G3: 68 ± 9 C: 67 ± 7	G1:25 (74) G2:35 (63) G3:59 (80) C: 33 (31)	G1: 47 ± 16 G2: 43 ± 16 G3: 42 ± 16 C: 50 ± 14	SWA	2 days	≥10 h	2 weekdays	Weekend days were excluded
Morita (2018) [30]	145	65 (60–73) median (IQR)	67 (46)	45 ± 15	Dynaport move-monitor	2 days	12 h	2 days	All days included
Schneider (2018) [35]	137	66 ± 8	75 (56)	46 (31–54) IQR	SWA	2 days	≥10 h	2 weekdays	All days included
**Studies that used a monitor without a thigh inclinometer and a waking hours protocol**									
Alyami (2018) [47]	34	62 ± 5	34(100)	46 ± 16	SAM	8 days	≥10 h	≥5 days	All days included
Bernard (2018) [38]	941	57 ± 15	519(55)	85 ± 28	Actical	7 days	≥8 h	≥4 days	All days included
Cruz (2014) [45]	16	66 ± 11	11(69)	70 ± 23	Actigraph	7 days	≥8 h	≥5 days	All days included
Cruz (2016) [46]	32	67 ± 8	27(84)	67 ± 20	Actigraph	4 weekdays	≥8 h	4 days	Weekend days were excluded
Eliason (2011) [48]	44	Mild COPD: 64 ± 6 Moderate COPD: 64 ± 8 Severe COPD: 63 ± 8	16(36)	Not recorded	Actigraph	7 days	≥8 h	≥3 days	All days included
Geidl (2019) [39]	326	58 ± 6	174 (68)	54 ± 18	Actigraph	7 days	≥10 h	≥5 days	All days included
Larson (2014) [50]	49	Ix: 71 ± 8 Ix: 72 ± 9 C: 71 ± 8	41(84)	Ix: 61 ± 20 Ix: 54 ± 17 C: 56 ± 17	Actigraph	7 days	≥10 h	≥3 days	All days included
Park (2013) [42]	224	70 ± 9	114(51)		Actigraph	7 days	≥10 h	≥4 days	All days included
Vasilopoulou (2018) [40]	147	Ix: 67 ± 10 Ix: 67 ± 7 C: 64 ± 8	Ix: 44(94) Ix: 38(76) C: 37(74)	Ix: 50 ± 22 Ix: 52 ± 17 C: 52 ± 21	Actigraph	Not recorded	≥8 h	≥4 days	All days included
**Studies that used a monitor with a thigh inclinometer and 24 h sleep removed protocol**									
Cheng (2020) [22]	69	74 ± 9	33 (48)	55 ± 15	ActivPAL	7 days	≥10 h	≥4 days	All days included
Hill (2020) [11]	11	72 ± 9	5 (45)	28 ± 26	ActivPAL	5 to 7 days	≥10 h	≥3 days	All days included
Mesquita (2017) [43]	90	67 ± 8	54 (60)	47 ± 9	MOX and CAM	≥7 days	≥10 h	5 days	All days included
Mesquita (2017) [44]	125	67 ± 4	69 (55)	50 ± 9	MOX	≥7 days	≥10 h	5 days	All days included
**Studies that used a monitor without a thigh inclinometer and a 24 h sleep removed protocol**									
Holland (2017) [9]	160	Ix: 69 ± 13 Ix: 69 ± 10	Ix: 48 (60) Ix: 51 (64)	52 ± 19 49 ± 19	SWA	7 days	≥10 h	≥4 days	Yes 1 w/e day included
Lewis (2016) [6]	24	75 ± 8	18(75)	54 ± 23	SWA + Actigraph	7 days	≥12 h	6 days	All days included
Loprinzi (2015) [51]	10	70 ± 10	4(40)	68 ± 48	Actigraph	7 days	≥10 h	4 days	All days included
McNamara (2014) [23]	50	COPD + PC: 73 ± 11 COPD: 70 ± 8	COPD + PC: 11 (44) COPD: 12 (48)	51 ± 17 54 ± 11	SWA	9 days	>85% wear time	3 days	All days included
Orme (2019) [49]	109	66 ± 7	67 (61)	76 ± 18	Actigraph	7 days	≥10 h	≥4 days	All days included
Schneider (2018) [36]	45	66 ± 8	25 (55)	46 ± 20	SWA	7 days	24 h (3 groups: 8 h; 12 h; period awake)	7 days	All days included
Wootton (2017) [10]	101	Ix: 69 ± 8 C: 68 ± 9	Ix: 38(61) C: 24(62)	Ix: 42 ± 15 C: 43 ± 15	SWA	7 days	≥12 h	≥3 days primary analysis ≥4 days secondary analysis	No (primary analysis); 1 w/e day included (secondary analysis)

Data are mean ± SD unless otherwise stated, A-MES: Activity Monitor and Evaluation System; C: control group; CAM: CIRO Activity Monitor; COPD + PC: chronic obstructive pulmonary disease + physical comorbidity; DAM: DynaPort Activity Monitor; FEV_1_% pred: forced expiratory volume in 1 s expressed as a percent predicted; Ix: intervention group; MOX: Mobile Only Accelerometer; O_2_: oxygen; SAM: Stepwatch Activity Monitor; SWA: SenseWear Armband.

## Data Availability

Detailed information on study data and analysis are available upon request from the corresponding author.

## Supplementary Materials

The following supporting information can be downloaded at: https://www.mdpi.com/article/10.3390/jcm11071980/s1, File S1: PRISMA checklist; File S2: meta-analysis output file; Figure S1: PubMed search strategy; Table S1: Data final.
