# Supplementary material for: Quantifying the Effect of Monitor Wear Time and Monitor Type on the Estimate of Sedentary Time in People with COPD: Systematic Review and Meta-Analysis
Source: J Clin Med. 2022 Apr 1;11(7):1980. doi: 10.3390/jcm11071980 (PMC8999633; doi:10.3390/jcm11071980)
Supplement: Supplementary file 1 [file jcm-11-01980-s001.zip › Figure S1. PubMed search strategy.pdf]

#1 Pulmonary disease, chronic obstructive [MeSH Terms]

#2 COPD [Title/Abstract]

#3 Emphysema [Title/Abstract]

#4 Chronic Bronchitis [Title/Abstract]

#5 Chronic Lung Disease [Title/Abstract]#6 #1 OR #2 OR #3 OR #4 OR #5

#7 Sedentary behavior [MeSH Terms]

#8 Sedentary behavio* [Title/Abstract]

#9 Sedentary lifestyle* [Title/Abstract]

#10 Sedentary time [Title/Abstract]

#11 Sedentariness [Title/Abstract]

#12 Physical inactivity [Title/Abstract]

#13 Physically inactive [Title/Abstract]

#14 Sitting [Title/Abstract]

#15 #7 OR #8 OR…#14

#16 #6 AND #1

Figure S1: PubMed search strategy
